# Changes in muscle oxygenation and activity during cumulative isometric muscle contraction: new insight into muscle fatigue

**DOI:** 10.3389/fphys.2025.1559893

**Published:** 2025-04-02

**Authors:** Junkyung Song, Yoon-Seok Choi, Sungjune Lee, Dawon Park, Jaebum Park

**Affiliations:** ^1^ Department of Physical Education, Seoul National University, Seoul, Republic of Korea; ^2^ Department of Kinesiology, Kyungpook National University, Sangju, Republic of Korea; ^3^ Advanced Institute of Convergence Science, Seoul National University, Seoul, Republic of Korea; ^4^ Department of AI-Integrated Education, Seoul National University, Seoul, Republic of Korea; ^5^ Institute for Sports Science, Seoul National University, Seoul, Republic of Korea

**Keywords:** surface electromyography, near-infrared spectroscopy, median-power-frequency, tissue oxygenation index, vastus lateralis muscle hemodynamics and EMG during repetitive-contraction

## Abstract

This study aimed to investigate the progression of muscle fatigue during submaximal efforts by examining alterations in muscle activation and oxygen saturation, employing surface electromyography (_*S*_EMG) and near-infrared spectroscopy (NIRS) measurements. Participants performed intermittent voluntary isometric knee extension tasks at 50% of maximal voluntary contraction to induce muscle fatigue. This was conducted consecutively until they could no longer generate the target torque. Knee extension torque, _*S*_EMG, and NIRS data from the vastus lateralis were collected. Torque variability, the magnitude and frequency of the _*S*_EMG signal, and NIRS-derived parameters of the tissue oxygen saturation index (TSI) were analyzed. An increase in the magnitude (*p* < 0.001) and a decrease in the spectrum (*p* < 0.001) of the _*S*_EMG signal were observed, followed by a rise in torque variability (*p* < 0.001), despite the average magnitude of knee extension torque remaining constant across the trials. The NIRS measurements indicated alterations in TSI parameters, reflecting increased metabolic demand and diminished oxygen supply in the fatigued muscle (*p* < 0.001). Furthermore, significant interrelationships were found between changes in torque, _*S*_EMG, and NIRS variables due to the development of muscle fatigue. Our findings provide a comprehensive understanding of the development of muscle fatigue, highlighting the interconnectedness of mechanical, electrical, and metabolic responses during submaximal efforts. The reduction in force-generation capacity due to muscle fatigue is reflected in the _*S*_EMG signal and manifests as an increase in motor variability. This study identified changes in the EMG and NIRS parameters, and significant interrelation between the two metrics during the process of fatigue accumulation. These findings have the potential to provide crucial knowledge for the prediction of fatigue from either EMG signal or hemodynamic signals of the muscles.

## 1 Introduction

Muscle fatigue is commonly defined as an exercise-induced reduction in the capacity to perform physical actions, often evidenced by a decrease in maximal muscle force or joint torque (Allen et al., 2008). It arises from impairments in various physiological factors crucial for enabling contractile proteins to generate mechanical output. These factors include alterations in neural contributions, regulation of excitation-contraction coupling, and various metabolic factors (Allen et al., 2008; Kent-Braun et al., 2012). Interestingly, in tasks demanding submaximal effort, which encompasses most activities of daily living, these physiological fatigue processes begin to manifest before any noticeable loss of force occurs (Gandevia, 2001; Sogaard et al., 2006; Enoka and Duchateau, 2008). The current study focused on this process of fatigue accumulation before reduction of mechanical output by examining changes during fatigue-inducing contraction.

The most evident phenomenon resulting from muscle fatigue was an increase in torque variability, even in participants capable of generating target torque (Contessa, Adam and De Luca, 2009). Furthermore, surface electromyography (_*S*_EMG) has been used to assess muscle fatigue (Farina et al., 2014). Changes in _*S*_EMG signals caused by muscle fatigue are measured in both the time and frequency domains. In the time domain, the integrated EMG (*i*EMG) is a commonly analyzed variable, reflecting the intensity of electrical activity in the muscle. However, the relationship between muscle force and EMG amplitude is not invariably linear in the fatigued condition. Indeed, the proportional relationship between muscle force and EMG amplitude is not sustained in the presence of muscle fatigue, as evidenced by the increase in EMG amplitude with diminished muscle force (Tabasi et al., 2023). This increase may be caused by the compensatory response to the reduced force-generation capacity of muscle fibers such as recruiting high-threshold motor units (Spiegel et al., 2012), synchronized activation of recruited motor units (Danna-Dos Santos et al., 2010) and metabolic changes in the fatigued muscle (Sahlin, 1992). In the frequency domain analysis, a shift of the median and mean power frequencies toward the lower band was observed (Cifrek, Medved, Tonkovic and Ostojic, 2009).

The functional role of the skeletal muscle as a physiological structure can be described using the metaphor of a mechanical motor that produces mechanical power while consuming gas and producing heat (Hill, 1938). The process of voluntary muscle activation is influenced by several factors, including neural drive, regulation of excitation-contraction coupling, metabolic processes, and heat, which is directly proportional to the mechanical work of the activated muscles (Allen et al., 2008; Kent-Braun et al., 2012; Allen, Lamb and Westerblad, 2008; Kent-Braun et al., 2014). Of particular relevance is the metabolic process within the skeletal muscle, which relies on oxygen as an energy source. Consequently, the near-infrared spectroscopy (NIRS) technique has been employed to quantify local oxidative metabolism in muscles during exercise. NIRS has been demonstrated to quantify the index of tissue oxygen saturation (TSI) by evaluating the proportion of oxygenated and deoxygenated hemoglobin within the muscles. This assessment has shown a reliable correlation to oxygen consumption, especially when the exercise intensity was low to moderate (Lucero et al., 2018). It has been postulated that several TSI parameters can reflect the magnitude and rate of oxygen desaturation during muscle contraction, as well as the extent of recovery post-contraction. These parameters have been demonstrated to provide reliably indicators of the level of muscular effort, as evidenced by studies reporting a correlation with joint torque (Sendra-Pérez C et al., 2023; Muthalib et al., 2010). The present study sought to address this research questions, “*Does the muscle require more or less oxygen when the magnitude of the mechanical outcome is reduced during the fatigue process*?” This research question has been partially addressed in previous studies. Specifically, a decline in the TSI and oxygenated hemoglobin levels during sustained force production has been observed, suggesting its potential as an indicator of the fatigue process (Keller et al., 2021; Keller et al., 2022). Building upon the findings of these earlier studies, the present experiment had two primary distinct objectives. Firstly, it sought to identify changes in NIRS parameters during the progression of fatigue. Secondly, it examined the relationship between NIRS parameters and other variables, such as torque and _*S*_EMG. Most previous studies utilizing NIRS to examine muscle fatigue have focused on sustained torque production tasks. The results of such experiments provide only information regarding the intensity or duration of the fatigue-inducing task. However, in daily activities, rather than fatigue accumulating due to sustained muscle contraction, it is more common for fatigue to gradually build up through repetitive actions which include both contraction and insufficient rest phases. A task in which the contraction phase and insufficient rest phase occur sequentially (Madeleine et al., 2002; Rashedi and Nussbaum, 2016), ultimately leading to exhaustion, allows for the examination of the fatigue and recovery process during repetitive torque production. While prior studies have utilized intermittent torque production protocol (Anders et al., 2021), the precise control of the rest phase in these protocols has been lacking. Consequently, the alternation in NIRS parameters during these repetitive rest phases remain ambiguous.

The purpose of this study is to investigate the changes in joint torque variability, muscle EMG signal and oxygenation during fatigue progression without a loss of mechanical output. Our first hypothesis was to confirm the induction of fatigue as manifested in the task used in the current study. We hypothesized that the development of muscle fatigue would lead to an increase in torque variability, even if the average torque magnitude remained constant. In addition, we expected that fatigue would be reflected by an increase in the *i*EMG and a decrease in the MDF, as quantified by the _*S*_EMG signals (Hypothesis 1). The second hypothesis was that the parameters of the TSI, measured by NIRS, would change systematically in response to the development of fatigue, reflecting the changes in oxygen demand and supply within the muscle tissue (Hypothesis 2). The third hypothesis focused on the relationship between mechanical outcomes, _*S*_EMG and NIRS variables. We postulated that these variables would be interrelated during the development of muscle fatigue (Hypothesis 3).

## 2 Materials and methods

### 2.1 Participants

Eleven male volunteers participated in this study. Their average age, weight, height, and thigh skinfold thickness were 26.74 ± 3.54 years, 73.55 ± 4.02 kg, 175.14 ± 3.76 cm, and 10.9 ± 2.1 mm respectively. All participants were right-leg dominance, as determined by their preferred leg for kicking a ball. None of the participants had a critical history of neuropathy or lower extremity trauma. In addition, it was confirmed that all the participants were able to perform the experimental task specifically designed for this study during participant screening procedures. According to the regulation of the Seoul National University Institutional Review Board, the experiment was conducted upon the approval of the proposed human experiment after the committee review along with IRB approval code (IRB No. 1712/001–006).

### 2.2 Experimental procedure

**FIGURE 1:**
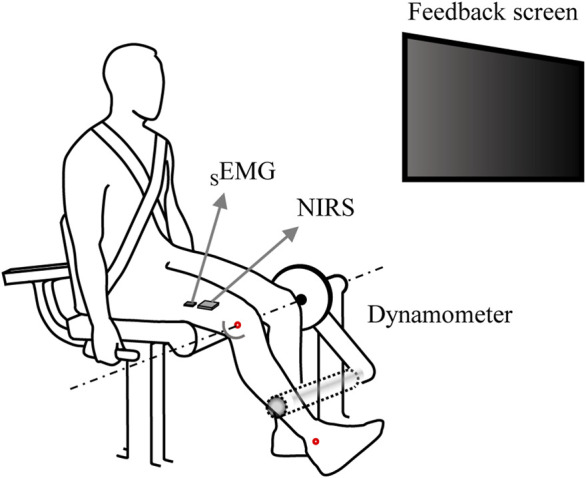
An illustration of the experimental setup. Participants positioned on the dynamometer with their right knee joint flexed at 60°, gripping the side handles firmly during the isometric knee extension torque production task. A monitor in front of the them provided real-time visual feedback on the knee extension torque. _*S*_EMG electrodes and NIRS sensors were attached to the belly of the vastus lateralis muscle, ensuring the measurement of joint torque, _*S*_EMG, and NIRS simultaneously.

Participants performed the intermittent voluntary isometric knee extension tasks using an isokinetic dynamometer (Cybex Humac Norm, CSMI, Stoughton, MA, USA) (Figure 1). They were seated on the dynamometer with its axis of rotation aligned with the right knee joint, using the lateral epicondyle of the femur as an anatomical reference (Maffiuletti et al., 2007; Kambič, Lainščak and Hadžić, 2020). The fully extended position of each participant’s right knee was defined as 0°, and then adjusted to a 60° flexion position. The lever arm was positioned 2 cm above the lateral malleolus of the right leg. The chest, hips, and thighs were secured to the dynamometer chair with Velcro straps while the side handle was held firmly to prevent excessive movement of body segments that were expected to contribute significantly to the measured knee extension value. The rotation axis of the knee was aligned with the mechanical axis of dynamometer, and the lever arm was positioned 2 cm above the lateral malleolus of the participants.

A wireless _*S*_EMG electrode (i.e., sensor) (Trigno Wireless EMG System, Delsys, Natick, MA, USA) and the NIRS sensor specifically designed to measure muscle oxygenation were used to measure the EMG and hemodynamic changes in the knee extensor muscle during the experimental protocol to induce muscle fatigue (Figure 1). A portable, dual-wavelength (780 and 850 nm) continuous wave muscle oxygenation monitor (REPACE, OBELAB, South Korea) was used to measure SmO_2_ responses. The device employed two LED light sources and two photodiodes, with multi source-detector distances (Choi and Han, 2019), and the modified Beer-Lambert law was used to account for light scattering.

The _*S*_EMG electrodes were attached to the belly of the vastus lateralis (VL) muscle of the right thigh using the manual palpation and SENIAM guidelines (Lee et al., 2022; Finni and Cheng, 2009), as the VL is known to be a primary representative muscle and shows the strongest linear relationship with mechanical outcomes (i.e., outcome force due to muscle contraction) among the quadriceps muscles (Alkner et al., 2000; Watanabe et al., 2018). The NIRS device was also attached to the VL, positioned more distally than the _*S*_EMG electrodes, at 75% of the distance from the anterior superior iliac spine to the lateral portion of the patella. Additionally, we confirmed by preliminary test that there was no signal interference between two devices. Knee extension torque, _*S*_EMG, and NIRS data were sampled at frequencies of 100 Hz, 2000 Hz, and 10 Hz, respectively, with all three measurement systems being physically synchronized. We achieved synchronization by providing a trigger pulse to three devices (force sensor, _*S*_EMG, and NIRS) using the customized LabVIEW program.

As a preliminary test, we first measured the maximum knee extension torque (MVIC_Torque_) of the participants’ right legs, which was then used to determine the target torque level for each participant to account for and normalize the difference in muscle strength between participants. The participants were required to reach the maximum joint torque within 5 s, and they performed two trials. We provided 3-min break between trials, and the average of the peak values from two attempts was used as MVIC_Torque_. The _*S*_EMG signals from the VL muscle were also measured to normalize the _*S*_EMG signals from the main tasks. After completing the MVIC_torque_ task, the participants took a break of approximately 10 min, followed by the main tasks. A few participants requested additional rest time, and we provided up to 30 min of rest.

The intermittent voluntary isometric knee extension task was to repeat the same magnitude of constant force production until the participant’s force production profile was deviated from the acceptable criteria. The criteria were set such that the measurement was stopped for a given episode if the torque generated by the participant fell below 90% of the target torque and could not be recovered within 5 s (Taelman et al., 2011). The level of target torque (force) was set at 50% of the MVIC_torque_. Note that multiple episodes of steady-state force production were performed in a single trial, and the number of the episodes could vary between the participants. The computer screen was positioned 1.2 m in front of the participant’s line of sight, displaying a horizontal bar along the x-axis to represent the target torque level. When the measurement began with the pop-up message on the computer screen, the participants were instructed to reach the target torque as accurately as possible within 1 s and to maintain the steady-state torque for 30 s. Participants were able to monitor their produced torque on the real-time feedback screen. Note that the measurement was not stopped at the end of the first episode, but the participants was asked to rest while maintaining initial posture for approximately 30 s. During the rest period, participants were instructed to relax completely; therefore, zero torque should be observed during the rest period. This duty cycle was determined through a preliminary test to progressively induce fatigue while ensuring that participants could sustain the task for multiple repetitions. The initiation and termination of each episode of force production was controlled by the experimenter’s verbal cue. For example, after 20 s of rest, the experimenter instructed participants to prepare for the next episode of torque production episode. The _*S*_EMG and NIRS data were collected simultaneously with the torque data during the intermittent voluntary isometric knee extension tasks. Due to the nature of the current experimental protocol, the total durations of the experimental time were varied, but the time variation between the participants was no more than ±10 min. The entire experiment, including orientation, experimental preparation, and the main test, lasted approximately less than 40 min for most participants.

### 2.3 Data analysis

**FIGURE 2:**
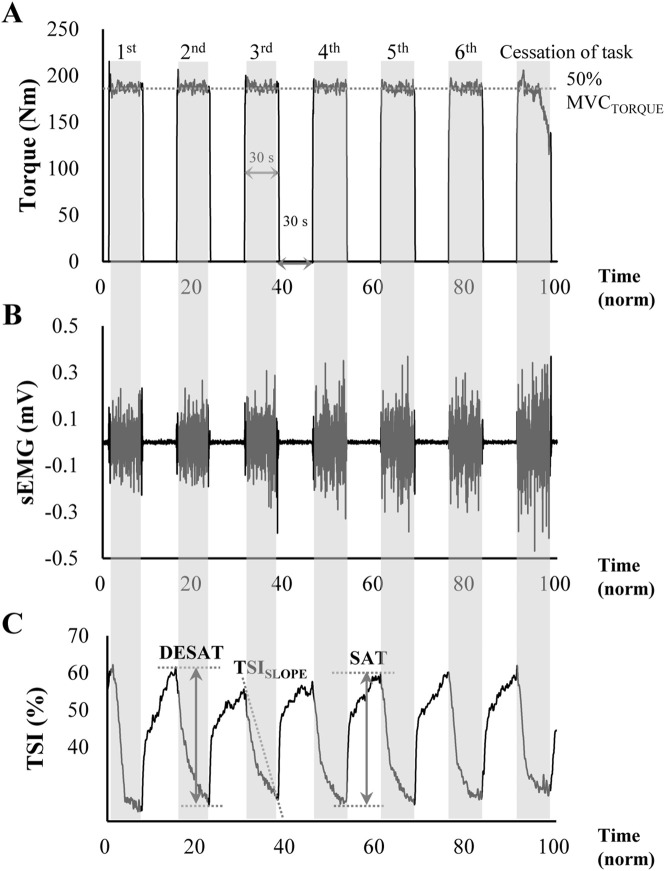
Representative data of **(A)** knee extension torque, **(B)**_*S*_EMG signal, and **(C)** tissue oxygen saturation index (TSI) from NIRS measurement during the intermittent voluntary isometric knee extension tasks to induce muscle fatigue. The target torque was set at 50% of maximal voluntary contraction, with participants generating a target torque for 30 s (shaded areas) followed by a 30 s rest period (non-shaded areas). The task was performed consecutively until participants could no longer produce more than 90% of the target torque. The ΔTSI of _DE_SAT represents the amount of oxygen desaturation shown in the shaded area, and the ΔTSI of SAT indicates the amount of oxygen saturation during relaxation. The TSI_SLOPE_ was calculated as the negative slope of the least squares regression line of TSI during the initial contraction period.

Three data sets, including torque, _*S*_EMG, and NIRS, were analyzed offline using customized code written in MATLAB (Math Works Inc., Natick, MA, USA). Within a single trial, multiple episodes were identified, with the understanding that the target torque was to be maintained at a consistent level, and the produced torque was to be larger than 90% of the target value on average over each episode. The first episode that deviated from the predefined criteria was classified as a failed episode. It should be noted that the data from these first episodes, as well as the failed episodes for each trial, were excluded from further analysis (see Figure 2A). In particular, the average torque of the last episode was less than 90% of the target torque, which was the criterion for ceasing the task. On average across participants, the number of episodes selected for the data analysis was 6.8 (±1.3 standard deviation, SD), ranging from 5 to 9.

#### 2.3.1 Resampling over a percentage of time

Because the number of episodes varied across participants, the following steps were taken to obtain comparable data set across participants: 1) Data from multiple episodes within a single trial from a given participant were aligned in chronological order. 2) The aligned data were resampled at 100 points (100% in time), excluding data from the first and last episode data. 3) The resampled data were divided into ***five time phases***, 0∼20, 20∼40, 40∼60, 60∼80, and 80%∼100%, and the average value within each phase was computed, resulting in all participants having a data set with the same number of phases consisting of five time sequence values. This approach was adopted to examine the progressive changes during the fatigue accumulation period in a normalized time domain, regardless of the number of episodes performed by different participants.

#### 2.3.2 Torque data

Torque data were filtered using a zero-lag fourth-order low-pass Butterworth filter with a 10 Hz cutoff. For the analysis segments of each trial, the average torque and the coefficient of variation (CV) were calculated separately over five phases for each participant.

#### 2.3.3 _S_EMG data

The primary analysis of the _*S*_EMG data was performed in the frequency domain. The data were first notch filtered at 60 Hz and then band-pass filtered between 20 and 450 Hz. For every 20 s of each trial, the filtered _*S*_EMG signals were transformed into a power spectrum, from which the median frequency (MDF) was calculated by identifying the frequency at the midpoint of the area under the power spectrum. In addition, the integrated EMG (*i*EMG) was calculated as an indicator of muscle activation intensity. This involved full-wave rectification of the _*S*_EMG signals within each data set, followed by envelope detection using a 100 ms window moving average filter. The resulting EMG data were integrated over 100 ms time windows to calculate the *i*EMG. Each participant’s *i*EMG was then normalized to the peak *i*EMG value obtained during the MVIC_torque_ tasks with the same time windows. We assumed that the electromechanical delay was about 50 ms (cf. Krishnamoorthy et al., 2003), and this value was applied to all trials for all participants.

#### 2.3.4 NIRS data

For the analysis of the NIRS data, spatially resolved spectroscopy method was used to estimate the tissue oxygen saturation index (TSI) (Baker et al., 2014; Song et al., 2020) (Equation 1).

$$TSI=\frac{{k\cdot HbO}_{2}}{k\cdot {HbO}_{2}+k\cdot HHb}\times 100$$ (1)

where *HbO*_*2*_, *HHb,* and *k* are the oxygenated, deoxygenated hemoglobin, and the constant scattering distribution respectively.

Figure 2C shows the typical TSI values in time series observed in the current experiment. As shown in Figure 2C, the TSI started to decrease rapidly when the muscle started its contraction, indicating oxygen desaturation within the muscle. In contrast, when the muscle was relaxed, the rate of the TSI recovery (i.e., the increase in TSI value) was relatively slow compared to the TSI change during the muscle contraction. The TSI-related variables (Muthalib et al., 2010; Linnenberg et al., 2023) included the changes in its magnitude (ΔTSI) during desaturation (_DE_SAT) and the saturation (SAT). Again, a larger ΔTSI of desaturation represents a greater oxygen demand relative to oxygen supply. Similarly, the amount of oxygen saturation during a resting period, ΔTSI of saturation, was quantified as the difference between the minimum TSI value and its maximum value at the end of the muscle contraction. The ΔTSI of SAT reflects the recovery capacity of the muscle, with an increase in ΔTSI of SAT indicating an increased oxygen supply relative to demand. Finally, TSI_SLOPE_, which represents the rate of oxygen desaturation in the initial contraction period, was calculated as the negative slope determined by the coefficient of the least squares linear regression.

### 2.4 Statistics

All descriptive statistics are presented as means with standard errors. To assess the changes in the dependent variables over a series of time phases, we performed one-way repeated measures ANOVAs with a factor of *Phase* (five levels: phase 1 ∼ 5). These analyses were applied separately to each variable, including mean torque, CV of torque, MDF, *i*EMG, and TSI_SLOPE_. For the statistical analysis of ΔTSI, the factor of saturation and desaturation phases, *TSI-DS* (two levels: SAT and _DE_SAT) was used for comparison along with the factor of *Phase*, which was performed by a two-way repeated measures ANOVA with factors of *Phase* and *TSI-DS*). Mauchly’s sphericity test was used to confirm sphericity assumptions, and violations were corrected by Greenhouse-Geisser estimation. Pairwise comparisons with Bonferroni correction were used for *post hoc* testing. The effect size, partial eta-squared (*ηp* ^*2*^), was calculated for all ANOVA tests. In addition, Pearson correlation analyses were performed to examine the relationships between torque-, EMG, and NIRS-related variables as they changed over time (phases). All statistical analyses were performed using SPSS version 25.0 (IBM, Armonk, NY, USA), and statistical significance was set at *p* < 0.05.

## 3 Results

In total, 11 participants were recruited for this experiment. However, one participant was excluded from the analysis due to muscle cramps during the experiment, and another participant was excluded due to fewer episodes than the predefined range. Therefore, the data from 9 participants were analyzed in the results.

### 3.1 Phase profile of torque production

**FIGURE 3:**
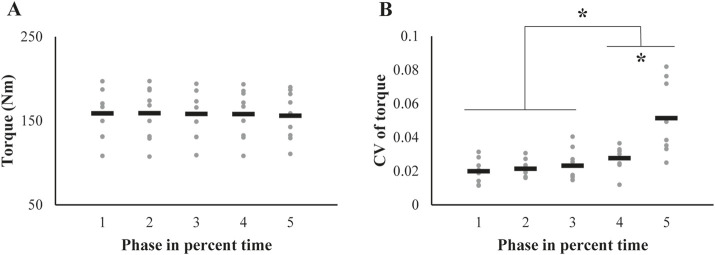
Changes in **(A)** the magnitude and **(B)** coefficient of variation (CV) of the knee extension torque across trials are represented by individual participant data (gray dots), with the mean values shown as black bars. The asterisk indicates significant changes from initial values, as determined by post-hoc analysis of the repeated-measures ANOVA.

The average torque magnitude across participants was nearly unchanged throughout the phases (Figure 3A), meaning that the outcomes of the other variables in the current analysis were estimated assuming the same torque magnitudes for all five phases. In contrast to the unchanged pattern of the torque magnitude, the CV of the torque increased at the later phase (Figure 3B). These findings were confirmed by a repeated-measures ANOVA, which confirmed a significant main effect of *Phase* on CV (*F*_[1.48,11.88]_ = 17.61, *p* < 0.001, *ηp* ^2^ = 0.69). Subsequent post-hoc pairwise comparisons confirmed the CV of **phase**1∼3 < 4 < 5 (*p* < 0.05).

### 3.2 Phase profile of EMG-related variables

**FIGURE 4:**
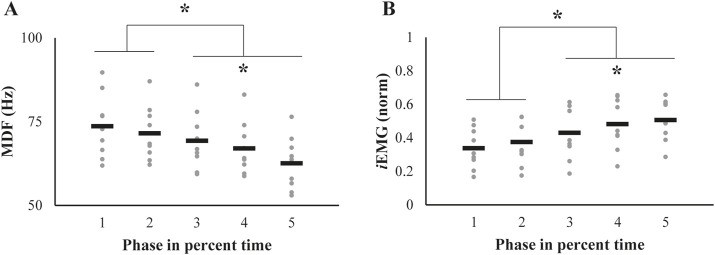
Alterations in **(A)** median frequency (MDF) and **(B)** integrated EMG (*i*EMG) throughout the successive fatiguing contractions. The data are represented as individual values for each participant (gray dots), with mean values indicated by black bars. Significant changes from their initial values, identified through post-hoc pairwise comparisons, are highlighted with an asterisk.

The two _*S*_EMG variables, median power frequency (MDF) and the integrated EMG (*i*EMG), showed opposite patterns of change over phases (Figure 4). The MDF showed the pattern of decrement (Figure 4A), whereas the *i*EMG values increased (Figure 4B). Repeated measures ANOVAs showed significant main effects of *Phase* on both the MDF (*F*_[1.59,12.7]_ = 25.9, *p* < 0.001, *ηp* ^2^ = 0.76) and *i*EMG (*F*_[1.99,15.97]_ = 32.35, *p* < 0.001, *ηp* ^2^ = 0.81). In addition, post-hoc analyses confirmed the MDF of **phase** 1 ∼ 2 > 3 > 4 > 5 (*p* < 0.05) and the opposite pattern of the *i*EMG of **phase** 1∼2 < 3 < 4 < 5 (*p* < 0.05).

### 3.3 Phase profile of NIRS-related variables

**FIGURE 5:**
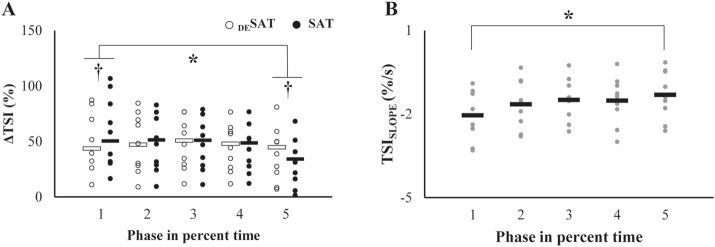
**(A)** Patterns of changes in the TSI (ΔTSI) across the trials. Each open and filled circle represents individual ΔTSI values of _DE_SAT and SAT, respectively, with the corresponding mean values indicated by open and filled bars. The asterisk denotes a significant change from the initial baseline. The daggers in the first and fifth trials indicate significant differences between the two parameters. **(B)** Changes in the TSI_SLOPE_ over repetitive trials. Each gray dot represents an individual participant’s value, and black bars indicate mean values. The asterisk indicates significant deviations from the initial value.

In general, ΔTSI of both saturation (SAT) and desaturation (_DE_SAT) decreased with the phases (Figure 5). A significant difference between the values of the phases was observed only between the phase 1 and phase 5 (Figure 5A). Accordingly, the significant differences between ΔTSI of SAT and _DE_SAT were also observed in the phase 1 and phase 5, which was supported by a repeated measures ANOVA with factors of *Phase (five levels)* and *TSI-DS (two levels:* SAT and _DE_SAT*)*, which showed a significant main effect of *Phase* (*F*_[1.74,13.94]_ = 17.32, *p* < 0.001, *ηp* ^2^ = 0.68) with a significant interaction of *Phase* × *TSI-DS* (*F*_[2.02,16.16]_ = 6.21, *p* = 0.01, *ηp* ^2^ = 0.44). The significant factor interaction reflected the fact that the ΔTSI of SAT was larger than that of _DE_SAT at the phase 1 (*p* < 0.05), whereas this contrast was reversed at the phase 5 (*p* < 0.05). In contrast to the decreasing pattern of the ΔTSI of _DE_SAT, the TSI_SLOPE_ increased significantly with phase (Figure 5B). Repeated measures ANOVA confirmed the significant main effect of *Phase* (*F*_[1.75,14.05]_ = 6.77, *p* = 0.011, *ηp* ^2^ = 0.46). Post-hoc pairwise comparison showed that TSI_SLOPE_ of **phase** 1 < 5 (*p* < 0.05).

### 3.4 Correlation analysis between the sets of variables: torque-, EMG- and NIRS-related variables

**FIGURE 6:**
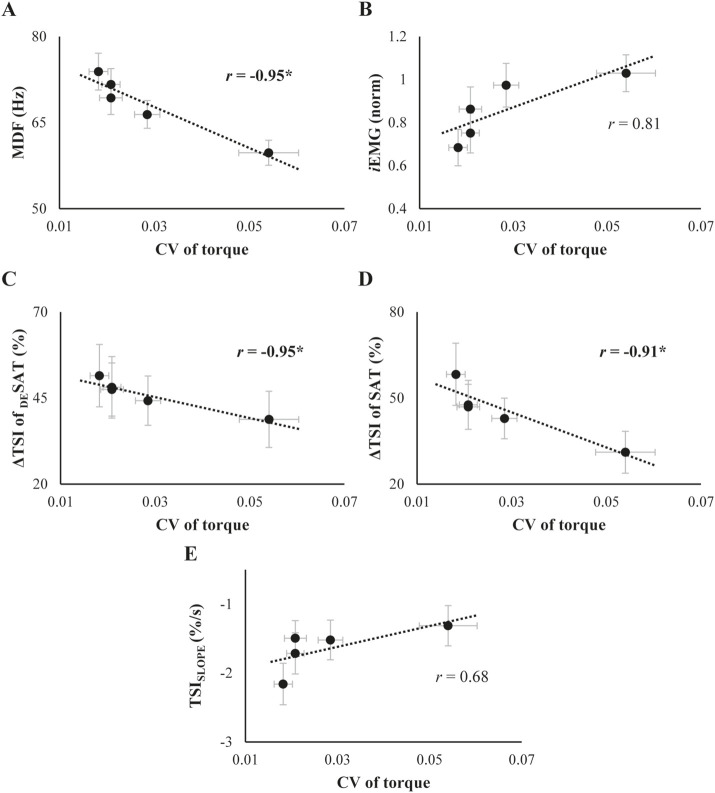
**(A)** and **(B)**: Relationship between the coefficient of variation (CV) of torque and SEMG variables. **(C-E)**: Relationship between the CV of torque and TSI parameters. The data represent the mean and standard error across subjects, collected throughout the trials. For each scatter plot, best-fit linear regression lines along with their correlation coefficients (*r*) are illustrated separately. The asterisk marks a significant correlation coefficient between the variables.

We further explored the relationship between changes in the torque-, EMG-, and NIRS-related variables whose main effects of *Phase* was significant. Changes in MDF showed a strong negative correlation with changes in CV of torque (*r* = −0.95, *p* = 0.012, Figure 6A). The CV of torque appeared to increase with change in *i*EMG, but this positive correlation was not statistically significant (Figure 6B). Regarding the relationship between the NIRS-related variables and CV of torque, ΔTSI changes were negatively correlated with CV of torque for both saturation (SAT) and desaturation (_DE_SAT) conditions (ΔTSI of _DE_SAT: *r* = −0.95, *p* = 0.015, Figure 6C; ΔTSI of SAT: *r* = −0.91, *p* = 0.033, Figure 6D). However, the changes in TSI_SLOPE_ did not show a significant relationship with the CV of torque (Figure 6E).

**FIGURE 7:**
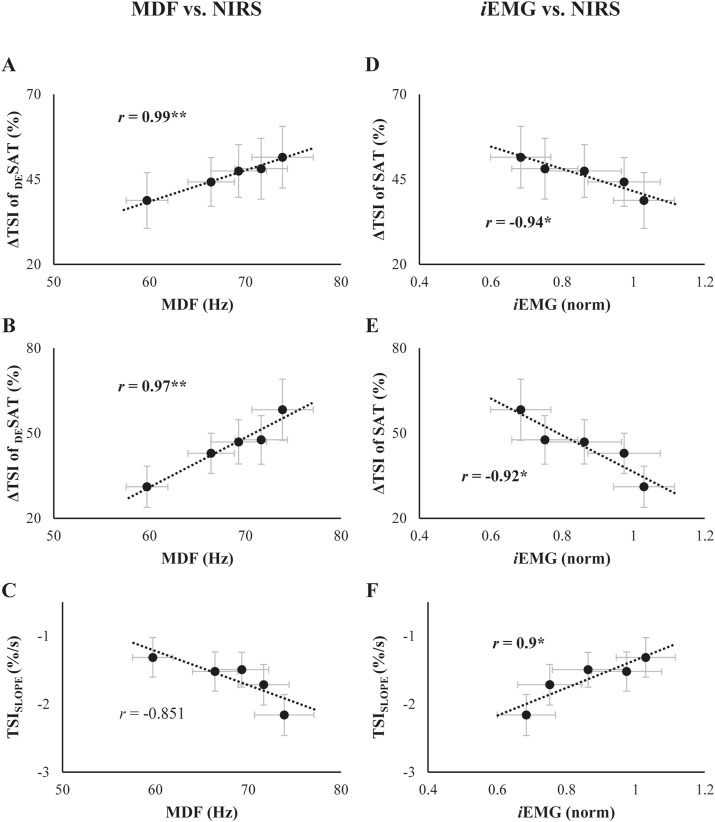
**(A-C)**: Relationship of the TSI parameters with median frequency (MDF) collected throughout the trials. **(D-F)**: Relationship of the TSI parameters with integrated EMG (*i*EMG) collected throughout the trials. Each scatter plot is presented in the order of the ΔTSI of _DE_SAT, ΔTSI of SAT, and TSI_SLOPE_, from top to bottom. The best-fit linear regression lines with coefficients of correlation (*r*) are presented. The asterisk indicates a significant correlation coefficient between the two variables.

The interrelationships between _*S*_EMG and NIRS variables are presented in Figure 7. Changes in the ΔTSI of both SAT and _DE_SAT conditions showed a significant positive correlation with the changes in MDF over phases (ΔTSI of _DE_SAT: *r* = 0.99, *p* = 0.001; ΔTSI of SAT: *r* = 0.97, *p* = 0.007, Figures 7A, B). Conversely, the TSI_SLOPE_ did not show a distinct correlation with MDF (Figure 7C). Furthermore, both ΔTSI of SAT and _DE_SAT negatively correlated with changes in *i*EMG (ΔTSI of _DE_SAT: *r* = −0.94, *p* = 0.018; ΔTSI of SAT: *r* = −0.92, *p* = 0.029, Figures 7D, E), while the TSI_SLOPE_ displayed a positive correlation with *i*EMG variations (*r* = 0.9, *p* = 0.038, Figure 7F).

## 4 Discussion

The main task of the current study was intermittent voluntary isometric knee extension task, which was intended to induce fatigue in the VL muscle, and its effect was expected to be cumulative until the torque profile deviated from the constrained condition. Note that the task was stopped at the episode (i.e., the last episode, not the sixth episode in Figure 2A) where the magnitude of the torque decreased by more than 10% of the target torque, and these data were excluded from the data analysis; therefore, the torque magnitudes of all five phases were expected to be the same, which was statistically confirmed. Furthermore, the current research questions and interpretations are at least independent from torque magnitudes. The curiosity underlying in the current hypotheses is whether fatigue is in the form of a threshold-like response (i.e., existence of minimum intensity of cumulative exercise required to induce muscle fatigue) or whether the phenomenon can be seen as a cascade of cumulative effects. The experiments substantiated all three specific hypotheses formulated in the Introduction. Specifically, the reverse pattern of the increased *i*EMG and the decreased MDF over muscle fatigue accumulation was confirmed (Hypothesis 1). The second hypothesis was confirmed by the finding that the TSI of both saturation (SAT) and desaturation (_DE_SAT) decreased with phases (i.e., fatigue cumulation). The third hypothesis was confirmed by the significant correlation between the NIRS parameters and EMG variables. In the Discussion, we address the implication of these findings for a number of issues, including the pattern of changes in muscle EMG signal and hemodynamics parameters when the muscle fatigue was cumulated by repetitive contraction.

### 4.1 Fatigue cumulation: cascading pattern of changes in muscle EMG signal

Based on the current results, the gradual accumulation patterns of the fatigue indices were observed. In fact, the variability of torque production, i.e., the coefficient of variation, showed a non-parallel change over the phases to its average magnitudes, which remained almost unchanged. There are many studies that have reported the increased variability with muscle fatigue (Enoka and Duchateau, 2008; García-Aguilar et al., 2022), so the current findings are not very new. However, we would like to highlight the current answer to the question “*when did these changes initiate?”*. The CV started to increase significantly from about the fourth phase, about 30 ∼ 60 s prior to the visible drop in torque magnitude, until the end of the last phase. Of course, the absolute time for this pattern would be different for different skeletal muscles, in part due to the compositional diversity of motor unit types within different muscles. Therefore, it would be a necessary and interesting future study to examine the timing difference between a set of muscles. Although the timing values could not be generalized without further investigation, we can now claim that the gradual increase in the outcome variability, i.e., the CV, would be considered as a precursor or monitoring index of muscle fatigue. Note that both non-linear changes, as demonstrated in this study, and linear changes of CV, as reported by Keller et al. (2021), have been reported. This discrepancy may be attributed to the dissimilarity of experimental setup and protocols between studies. Given that CV is a straightforward yet conspicuous variable for detecting and monitoring muscle fatigue, future studies must examine the causes of linear and non-linear changes in CV during muscle fatigue.

Other results from the EMG-related variables were also consistent with previous findings from EMG data analysis (Ertl et al., 2016). In particular, the median power frequency (MDF) and the integrated EMG (*i*EMG) showed gradual changes before noticeable fatigue, while the change patterns of the two variables were opposite, showing decreasing and increasing patterns of the median power frequency (MDF) and the integrated EMG (*i*EMG), respectively. It has been well reported that the spectral parameters of the _*S*_EMG shift towards a low frequency band due to relatively slower muscle fiber recruitment and a reduction in muscle fiber conduction velocity during the accumulation of muscle fatigue development (Farina, 2006).

To summarize the EMG-related variables with the performance, as muscle force-generating capacity declines, it is highly likely that the neuromotor system adapts by modulating the recruitment and synchronization of motor units to maintain mechanical output even when the muscle fatigue was accumulated by the repetitive contraction (Pethick et al., 2021). These adaptations, as evidenced by the volume of muscle activation, i.e., *i*EMG, and a decrease in median frequency (MDF), support our first hypothesis (Figure 4). It was noted that significant changes in _*S*_EMG variables preceded the increase in the CV of torque, which became more pronounced just prior to task failure. Combined with the significant correlation found between these variables (Figure 6A), the novel finding of the current experiment is that altered muscle activation patterns due to cumulated fatigue could have a detrimental effect on the stability of muscle force production (Cifrek et al., 2009). Furthermore, the results observed in the torque and _*S*_EMG variables indicate that the submaximal contraction task employed in this study successfully induced muscle fatigue.

### 4.2 Fatigue cumulation: inversion patterns of the TSI during oxygen saturation and desaturation of the muscle

In the analysis procedure for the NIRS parameters, TSI-related variables were mainly implemented, rather than oxygenated and deoxygenated hemoglobin data. The inherent limitation of NIRS measurement is that the measured oxygenated and deoxygenated hemoglobin are not absolute values but relative values (i.e., changes), which usually have large variability, leading to compromised normality in data distribution. While the TSI values were also estimated using relative values, it is crucial to note that the TSI value was the normalized values with respect to the total hemoglobin. This modification ensures that the comparisons of TSI values across conditions would be reliable in statistical analyses.

It appears that the tissue oxygen saturation index (TSI) measured by the NIRS device showed a rapid decrease at the beginning of the muscle contraction (*see* larger values at phase 1 in Figure 5A), followed by relatively subtle changes until the end of the muscle contraction (Figure 2). One possible interpretation of the TSI change, i.e., decrease, during muscle contraction is that the restricted blood flow associated with elevated intramuscular pressure by muscle contraction may cause an imbalance between oxygen supply and consumption such that the relatively increased oxygen consumption compared to the resting state of the muscle under the condition of limited oxygen supply (Rissanen et al., 2012; Solsona et al., 2021; Song et al., 2020). Nevertheless, it must be acknowledged that the NIRS device’s capacity to assess blood flow is inherently constrained. Consequently, the present interpretation remains provisional and is slated to be addressed in forthcoming studies employing apparatus capable of more precise blood flow estimation. It has been suggested that the hemodynamic changes caused by muscle contraction represent an imbalance between oxygen demand and supply, and there is experimental evidence that this imbalance is critically linked to muscle fatigue (Yoshitake et al., 2001). Our second hypothesis was that the parameters of the TSI would change during repetitive contractions, reflecting the accumulation of muscle fatigue. We observed a decrease in ΔTSI of _DE_SAT and ΔTSI of SAT and an increase in the slope of TSI (TSI_SLOPE_) over successive trials, although the magnitude of torque was maintained (Figure 5). ΔTSI of _DE_SAT and ΔTSI of SAT reflect the balance between oxygen supply and consumption of muscle tissue during contraction and relaxation, respectively. TSI_SLOPE_ quantifies the rate of oxygen desaturation during the initial phase of muscle contraction. It has been observed that these parameters of TSI are highly correlated with the intensity of muscle contraction (Felici et al., 2009; Muthalib et al., 2010; Song et al., 2020). Therefore, it can be inferred that the gradual changes in these parameters while maintaining the output torque could imply a reduced force production capacity in the contracting muscle due to reduced oxygen utilization and increased oxygen debt. This could potentially lead to energy replenishment via anaerobic glycolysis and lactic acid accumulation, which might further exacerbate muscle fatigue (Westerblad et al., 2002). This could result in the inversion of the magnitudes of ΔTSI of SAT and _DE_SAT at the last phase just before visible drop of torque production capability (phase 5 in Figure 5A). However, the studies by Brooks G. et al. (2006) claimed that the traditional view of the lactic acid as a cause of fatigue is not always true. In other words, lactic acid could be a source of fuel by transporting to other structure; thus, the causes of muscle fatigue contain multifactorial components (Brooks et al., 2006). Therefore, the aforementioned potential mechanism may not be a sole factor for the muscle fatigue in the current experiment.

### 4.3 Mechanism of muscle fatigue cumulation

The comparison of the indices of muscle fatigue between the indices from the well-established approach, e.g., estimation of changes in the MDF and *i*EMG, and from the measurement of hemodynamics during muscle contraction was not just for examining significant relationship between two sets of measures in statistics. Of course, a simple interpretation would presumably provide useful information as to the possibility of “*interoperability*” between the EMG and NIRS measures to identify the muscle fatigue. In this regard, one of the notable observations was the significant correlation observed between changes in NIRS parameters and both torque variability and _*S*_EMG variables (Figures 6, 7). This supported our third hypothesis and highlights the interconnectedness of mechanical, electrical, and metabolic responses during the development of muscle fatigue, suggesting that NIRS parameters could be a possible indicator to evaluate the fatigue process. Consequently, the two metrics from the muscle activation would be commonly useful for monitoring the fatigue process during sustained and repetitive contraction. It is imperative to discern the distinguishing features between NIRS- and EMG-related variables in order to effectively detect muscle fatigue. It is well established that the hemodynamic and metabolic responses are known to be slower than the EMG response, which can be attributed to the disparate response times of electrical activity and metabolic changes (Daniel et al., 2023; Sun et al., 2022; Yoshitake et al., 2001). The current results of early changes in EMG variables and delayed changes in TSI parameters are consistent with previous findings. Moreover, it is plausible to hypothesize that the observed discrepancy between the two metrics in terms of response time can be applied not only to the acute time scale (i.e., acute response) but also to repetitive contraction in the relatively larger time scale.

Lastly, ΔTSI of _DE_SAT and ΔTSI of SAT, which are related to the magnitude of oxygen demand and supply, showed a higher correlation with torque and _*S*_EMG variables compared to TSI_SLOPE_, indicating that these parameters might serve as more appropriate indicators of fatigue development. We further observed that _*S*_EMG variables began to change early in response to fatigue accumulation, whereas TSI parameters demonstrated significant alterations closer to the phase of task failure (i.e., the fifth trial). This implies that these parameters may serve as predictive markers for the point of muscle exhaustion during submaximal effort. It should be addressed in more detail in future research.

One of the primary limitations of this study is the relatively small sample size, which may limit the generalizability of our findings. However, despite this limitation, we observed large effect sizes in most key variables, suggesting meaningful trends in the data. Nevertheless, future studies with larger sample sizes are needed to further validate our findings and strengthen the statistical reliability of these results.

## 5 Conclusion

This study explored the progression of muscle fatigue during submaximal efforts, examining changes in muscle activation and oxygen saturation through the integration of _*S*_EMG and NIRS measurements. We confirmed that fatigue-induced variations in the magnitude and spectrum of _*S*_EMG signals occurred prior to an increase in torque variability, even as the average torque magnitude was maintained. This could be a reflection of compensatory adjustments to the decreased capacity for muscle force production, attributed to the accumulation of fatigue from repetitive torque production tasks. It is interesting to note that TSI parameters, as measured by NIRS, responded to the development of fatigue, reflecting a diminished oxygen utilization capacity in the contracting muscle. This observation underscores the potential of these parameters as indicators of muscle fatigue during intermittent submaximal efforts. The significant correlations found between torque, _*S*_EMG, and NIRS variables shed light on the complex nature of muscle fatigue. Overall, utilizing NIRS’s capability for continuous monitoring of active muscles not only provides insights into oxygen saturation but also presents a valuable tool for assessing muscle fatigue development, including predicting potential muscle exhaustion critical for injury prevention.

## Data Availability

The raw data supporting the conclusions of this article will be made available by the authors, without undue reservation.
